# Contribution of indigenous foods towards nutrient intakes and nutritional status of women in the Santhal tribal community of Jharkhand, India

**DOI:** 10.1017/S1368980016000318

**Published:** 2016-03-16

**Authors:** Suparna Ghosh-Jerath, Archna Singh, Melina S Magsumbol, Tanica Lyngdoh, Preeti Kamboj, Gail Goldberg

**Affiliations:** 1 Indian Institute of Public Health–Delhi, Public Health Foundation of India, Plot no. 47, Sector 44, Institutional Area, Gurgaon 122002, India; 2 All India Institute of Medical Sciences, New Delhi, India; 3 Public Health Foundation of India, Gurgaon, India; 4 Nutrition and Bone Health Research Group, MRC Human Nutrition Research, Elsie Widdowson Laboratory, Cambridge, UK

**Keywords:** Indigenous foods, Santhal tribes, Nutrient intake, Nutritional status

## Abstract

**Objective:**

The indigenous food environment, dietary intake and nutritional status of women in the Santhal tribal community of Jharkhand were assessed. Contribution of indigenous foods to nutritional status and nutrient intakes was explored.

**Design:**

Exploratory cross-sectional study with a longitudinal dietary intake assessment component. Household and dietary surveys were conducted to elicit information on socio-economic and demographic profile and food consumption patterns at household level. A 24 h dietary recall for two consecutive days (repeat surveys in two more seasons) and anthropometric assessments were carried out on one woman per household.

**Setting:**

Households (*n* 151) with at least one woman of reproductive age in four villages of Godda district of Jharkhand, India.

**Subjects:**

Women aged 15–49 years.

**Results:**

Almost all households owned agricultural land and grew fruits and vegetables in backyards for household consumption. A wide variety of indigenous foods were reported but dietary recalls revealed low intake. Women consumed adequate energy and protein but micronutrient intake was inadequate (less than 66 % of recommended) in the majority (more than 50 %) for Ca, Fe, vitamin B_2_, folate and vitamin B_12_. Women consuming indigenous foods in the past 2 d had significantly higher intakes of Ca (*P=*0·008) and Fe (*P=*0·010) than those who did not. Varying degrees of underweight were observed in 50 % of women with no significant association between underweight and consumption of indigenous foods.

**Conclusions:**

Promotion of preferential cultivation of nutrient-dense indigenous food sources and effective nutrition education on their importance may facilitate better micronutrient intakes among women in Santhal community of Jharkhand.

India is home to a large number of indigenous tribal people, who are still largely untouched by globalization and its impact on conventional lifestyles^(^ ^1^ ^)^. These indigenous communities live in a rich habitat replete with food resources and practise deep-rooted knowledge of the forest environment. However, despite this environmental endowment, challenges of geography, limited use of agricultural technology, cultural habits, lack of formal education, poor access to health-care facilities and poverty have resulted in poor nutrition and health in these communities.

Consumption of indigenous wild foods like wild plant foliage, vegetables and fruits can be an important mode of dietary diversification and a direct food-based intervention to address food insecurity^(^ ^2^ ^,^ ^3^ ^)^. Apart from foraging, indigenous communities in India are often small farm holders who cultivate plots of land or backyard gardens (also known as kitchen gardens). Green leafy vegetables are grown in these kitchen gardens and are used for everyday cooking. Indigenous crops are seen to contain more micronutrients than non-native species of vegetables and promotion of indigenous crops is shown to be integral for ensuring that households consume more diverse diets^(^ ^4^ ^)^. Various traditional leafy vegetables native to specific regions promoted by home gardening programmes have shown a positive impact on food and nutrition security. Specifically, such home garden interventions with or without vitamin supplementation strategies have resulted in a substantial decline in vitamin A deficiency^(^ ^5^ ^)^.

Other studies have also reported that regular consumption of indigenous varieties of fruits and vegetables in communities could potentially fulfil dietary recommendations for various micronutrients like vitamin A, Ca and trace elements^(^ ^6^ ^–^ ^11^ ^)^. Further, increased consumption is also indicated as a strategy for improving rural economies by increasing their production and assisting in the creation of markets for these foods. Food quantity and quality are the most direct indicators of the synergy between public health nutrition, agriculture and food security; understanding the extensive diversity and complexity of food systems of indigenous peoples and improving and strengthening these systems in the context of nutrition and health thus merit attention^(^ ^12^ ^)^.

In spite of huge natural resources, Jharkhand, a state in the central eastern part of India, is a poor performing state with some of the worst nutrition indicators. The prevalence of underweight and anaemia among women in the state is 42·6 % and 70·6 %, respectively^(^ ^13^ ^)^. The state is home to thirty-two Scheduled Tribes. The Santhal communities are the most populous, comprising about a third of the indigenous population in the state^(^ ^14^ ^)^. Studies have reported a high prevalence of undernutrition, chronic energy deficiency (CED) and Fe-deficiency anaemia in adults and children of the Santhal community residing in different states of India, including Jharkhand, Orissa and West Bengal^(^ ^15^ ^–^ ^19^ ^)^. This is despite a rich inventory of indigenous foods of both plant and animal origin in these states^(^ ^20^ ^)^. Although there are studies in India that document the nutritive value of indigenous foods consumed by tribal groups, there is a paucity of data on quantitative consumption estimates of actual intakes of these foods and their contributions to recommended dietary intakes^(^ ^21^ ^–^ ^23^ ^)^. It is imperative that health scientists and nutrition researchers explore and conduct empirical research on the potential of traditional food systems in increasing dietary diversity and contributing to food security^(^ ^24^ ^)^. This is also relevant in the context of current efforts globally that are looking at ways to increase consumption of local foods as well as for conserving biodiversity and indigenous knowledge, both within the community and as a knowledge resource for the scientific community. To the best of our knowledge, no study among Santhal tribal communities in India has explored the contribution of indigenous foods to nutrient intakes and their potential role in addressing food security.

The present study is a part of larger project that investigated the nutrient composition of common indigenous foods consumed by two tribal communities of Jharkhand and their contribution to food and nutrition security of women and children in the study communities. In the present paper we document our findings on the contribution of indigenous foods to nutrient intakes and nutritional status of women in the Santhal tribal community of Jharkhand.

## Methods

### Study design

This was an exploratory, cross-sectional survey with a longitudinal component on dietary intake assessment to account for seasonal variations.

### Study settings

Four villages in the Sunderpahari block of Godda district of Jharkhand were covered.

### Study period

The data were collected in three seasons. In June 2013 (rainy season), a detailed household (HH) and dietary survey was conducted. This was supplemented with a 24 h dietary recall (DR) on two consecutive days of one woman of reproductive age (15–49 years) per HH. A third of the same women were followed up in November 2013 (winter) and May 2014 (summer) by undertaking a two-day 24 h DR to capture seasonal variations in food and nutrient intakes.

### Sample size calculation

In order to assess CED (BMI<18·5 kg/m^2^) in the study community, the objective of the sampling was set to capture at least 40 % prevalence of CED^(^ ^13^ ^)^. With *α* level of 5 %, design effect of 1·5 and an error range of ±10 % with 95 % confidence, a sample size of 140 was arrived at. We also calculated the sample size based on the proportion of the Indian rural population who are projected to be consuming inadequate energy (which is 62 %)^(^ ^25^ ^)^ and the prevalence of micronutrient deficiency in women (Fe-deficiency anaemia, 59 %)^(^ ^13^ ^)^. The sample size estimates using these different assumptions were the same.

### Sampling framework

Based on the estimated sample size, 151 HH were selected from the chosen block in Godda district with a high concentration of Santhal population. A total of four villages were identified using probability-proportional-to-size sampling^(^ ^26^ ^)^ and at least thirty-five HH were selected from each village. Since the main study aimed to look at both maternal and child undernutrition, HH surveyed were those with at least one child under the age of 5 years. These HH were selected randomly from the list of eligible HH provided by the *anganwadi* centre (community-based maternal and child health and nutrition care centre where every child under 5 years is enrolled) catering to the villages. To ensure random selection of at least thirty-five HH, additional HH were surveyed from adjacent villages inhabited by Santhal tribes if a village had less than seventy HH with children under the age of 5 years. One woman of reproductive age from each HH was selected using the Kish selection table (a method for randomly selecting members within a HH to be interviewed)^(^ ^27^ ^)^ for the purpose of the two-day 24 h DR and anthropometric assessment.

### Data collection

Quantitative methods were used for data collection. An interviewer-administered questionnaire was used. This questionnaire was pre-tested in two Santhal villages. Information was collected on HH socio-economic and demographic profile, food consumption patterns with emphasis on the intake of indigenous food, procurement of food and money spent on food. HH food security was assessed using questions from the Household Food Security Scale–Short Form developed by the US Department of Agriculture^(^ ^28^ ^,^ ^29^ ^)^. The frequency of consumption of different food groups was assessed using an FFQ. Pre-survey visits and qualitative enquiries through key informant interviews and focus group discussions were conducted to capture the variety of foods consumed by the Santhal community and their knowledge of the nutritional and medicinal properties of these items. Food items identified during the focus group discussions were extensively used to develop the FFQ. We developed a Santhal FFQ, pre-tested it in two villages with Santhal tribal communities and expanded the list of food items to a 243-item FFQ. These included both commonly consumed Indian food items as well as indigenous tribal foods. The reference period for the FFQ was 1 month. The field workers were provided extensive training including hands-on training in the field. This FFQ was administered during the survey conducted in the rainy season.

A two-day 24 h DR was taken for one woman of reproductive age per HH. For the DR, the selected woman was asked by an interviewer to remember, in as much detail as possible, her food intake during the past 24 h by using a food recall kit. For each meal, the respondent was asked to recall and wherever possible show or describe the foods eaten (i.e. each food item consumed along with a detailed recall of ingredients used, method of preparation, etc.) based on the recipe. A food recall kit which included standard utensils, measuring cups, spoons, glasses and a weighing scale was used for this purpose. This exercise of eliciting a 24 h DR was repeated on one-third of the population in two more seasons. So, data were collected during three seasons (summer, rainy and winter) in order to capture seasonal variations in nutrient intakes. Prior to the dietary survey, the study team had explored the availability and utilization of commonly consumed indigenous foods in the community. These commonly consumed foods were collected from the field and sent for taxonomic classification. Based on their scientific names, they were looked up in the Indian food composition tables called *Nutritive Value of Indian Foods* ^(^ ^30^ ^)^. Efforts were made to collect samples of those food items that were reported in the DR but for which nutritive values were not available. These food samples were sent for nutrient analysis in an NABL (National Accreditation Board for Testing and Calibration Laboratories) certified laboratory in Delhi. Nutrient analysis was done using standard protocols and methodology. Mandatory quality control and quality assurance procedures of the laboratory were followed during analysis of each nutrient. The nutritive values of these foods were then entered into the software that was used for conducting nutrient analysis of the DR.

Anthropometric assessments to measure height and weight were carried out using standard protocols and equipment (weighing scale and anthropometer rod) on the same woman from whom DR were taken.

All study tools were translated by a native speaker into the local language (Santhali) and back-translated into English to check for fidelity, accuracy and consistency. The study tools were in the native language and administered by the field workers who were well versed in the local language/dialect. These field workers were accompanied by the study team members who supported them in eliciting accurate information from the study participants.

### Data analysis

Nutrient intake data from the DR were entered into the validated software ‘DietCal’ version 3·0 (Profound Tech Solution; http://dietcal.in/), which is based on values from the *Nutritive Value of Indian Foods* ^(^ ^30^ ^)^. Nutrient intake data (as represented by mean) were then compared with the *Recommended Dietary Allowances for Indians* for a moderately active adult woman^(^ ^31^ ^)^. The adequacy of nutrient intake by each participant was computed in terms of the nutrient adequacy ratio (NAR)^(^ ^32^ ^)^:

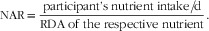

Cut-offs for NAR were considered as ‘inadequate’ when the ratio was less than 0·66; ‘fairly adequate’ when the ratio was 0·66 to <1·00; and ‘adequate’ when the ratio was ≥1·00.

For the HH food security scores, the sum of affirmative responses to the six questions in the Household Food Security Scale–Short Form provided the HH’s raw score on the scale. Food security status was then classified based on the raw score: 0–1 as high or marginal food security (raw score=1 may be considered as marginal food security); 2–4 as low food security; and 5–6 as very low food security. BMI was calculated as weight (in kilograms) divided by the square of height (in metres). The women were classified as being underweight and as having CED or not using standard BMI cut-offs^(^ ^33^ ^)^. In order to assess any misreporting of dietary intake data in the context of energy intake estimation, we calculated Goldberg’s cut-off^(^ ^34^ ^)^ for mean ratio of energy intake to BMR at a group physical activity level (PAL) of moderately active women.

Continuous variables were summarized as mean and standard deviation or as median and interquartile range, while categorical variables were summarized as number of subjects and percentages.

Women were categorized into two groups: those who consumed and those who did not consume indigenous foods based on 24 h DR data. The nutrient intakes in the two groups were then compared. Similarly, the nutritional status of women was compared between the groups. We used the *t* test or Wilcoxon rank-sum test and the *χ* ^2^ test to compare the differences in distribution of continuous and categorical variables, respectively, between the two groups. Seasonal variation in nutrient intakes was identified using ANOVA and the pairwise *t* test was done after Bonferroni correction using a *P* value of 0·02 (i.e. 0·05 divided by the number of comparisons, in this case three) to identify where differences existed between groups. This was performed if the *F* value in ANOVA was significant. We used two-sided tests and considered statistical significance at *P*<0·05. Analyses were performed using the statistical software package STATA version 13.

## Results

Thirty-six to thirty-nine HH each in the four villages of Mahuatand, Teelabad, Bariyarpur and Kadampur in the Sundarpahari block of Godda district of Jharkhand, belonging to Santhal tribal community, were covered. Table 1 shows the socio-economic and demographic profile of HH as well as their food security status. The ratio of males to females in the study population was 0·83. The mean household size was 5·5 (sd 1·8; range: 2–12). Adult women comprised 28·04 % of the population (*n* 831). The mean age of the reproductive-age women was 27·8 (sd 7) years.

**Table 1:** Socio-economic and demographic profile of the sampled households in the Santhal tribal community of Jharkhand, India

Variable	Category	*n*	%
Family type	Nuclear	86	57·0
(*n* 151)	Joint	46	30·5
	Extended	19	12·6
Religion	Hindu	131	86·8
(*n* 151)	Christian	20	13·2
Literacy level of household head	Illiterate	92	61·3
(*n* 150)	Can read and write	27	18·0
	Schooling (primary, middle, high)	29	19·3
	Graduate	1	0·7
	Studying	1	0·7
Occupation of household head	Unemployed	1	0·7
(*n* 150)*	Help in household chores	7	4·7
	Daily wage earner (agriculture)/farmer	121	80·7
	Daily wage earner (non-agriculture)	10	6·7
	Artisan	1	0·7
	Small business owner	2	1·3
	Housewife	4	2·7
	Service (non-government and government)	4	2·7
Literacy level of all reproductive-age women	Illiterate	145	73·2
(15–49 years) in household	Can read and write	35	17·7
(*n* 198)	Schooling (primary, middle, high)	13	6·6
	Studying	5	2·5
Family income (INR)	<1000	63	41·7
(*n* 151)	1000–2000	47	31·1
	2000–5000	33	21·9
	5000–10 000	6	4·0
	>10 000	2	1·3
PDS ration card†	Red (BPL‡)	30	19·9
(*n* 151)	Yellow (AAY§)	5	3·3
	Don’t have	116	76·8
Household food security	High food security	19	12·7
(*n* 150)*	Low food security	41	27·3
	Very low food security	90	60·0

* Missing data on socio-economic and demographic profile of the households ranged from 0·7 to 7·9 %.

† The Public Distribution System (PDS) is an Indian food security system under the Ministry of Consumer Affairs, Food, and Public Distribution. In this, major food commodities including staple food grains (such as wheat and/or rice), sugar and kerosene oil (a fuel used for cooking) are distributed through a network of public distribution shops (also known as ‘ration shops’) at subsidized prices. The PDS ration card is an official document entitling the holder to a ration of food under various categories of poverty.

‡ Below the poverty line.

§ AAY (*Antyoday Ann Yojana*), a category based on degree of poverty, entitles the holder to access food products at highly subsidized prices.

### Land ownership for agriculture and access to food

Santhal communities are small landholder farmers; almost all HH (97·4 %) owned land for agricultural purposes. The majority of HH (97·3 %) utilized the food they grew for household needs. The HH (90·7 %) also utilized their backyard gardens, popularly called *bari*, for growing vegetables and fruits which were mostly (96·4 %) used for household consumption. Most of the participating HH (76·8 %) owned livestock: goats, sheep, cattle, poultry and pigs. A quarter of the food consumed by the majority (41·6 %) of HH was either home-produced food and/or procured from nearby forests. Foods that were procured from market or *haat* (weekly local market) included rice, oil and sugar, on which money spent per month (*n* 150) was INR 1279·3 (sd 968·9; range: INR 100–5000). Most of the HH (60·0 %) had very low food security. In spite of this, the access to food security programmes as observed through possession of a PDS (Public Distribution System) ration card (a government programme addressing food security for socio-economically disadvantaged households or those belonging to various grades of poverty) was low (about 23 %) in the study community.

### Consumption and production of indigenous foods

The present study focused predominantly on eliciting information about the consumption and contribution of indigenous foods to the dietary intake of Santhal women. The consumption of a variety of indigenous foods including green leafy vegetables (GLV; 100·0 %), meat and meat products (98·7 %), fruits (98·7 %), vegetables (98·0 %) and roots and tubers (92·1 %) during specific seasons or throughout the year was reported by majority of HH. Indigenous GLV mostly consumed included (local name (scientific name)): *gandhari* (*Amaranthus spinosus*); *munga arak* (*Moringa oleifera*); *kantha arak* (*Euphorbia granulate*); *sin arak* (*Bauhinia purpurea*); *dhurup arak* (*Leucas cephalotes*); *haisa arak* (*Ficus religiosa*); *matha arak* (*Antidesma diandrum*); *turi arak* (scientific name not known); *lapaung arak* (*Areua lanata*); *ohio arak* (*Boerhaavia diffusa*); *garundi arak* (*Alternanthera sessilis*); *but arak* (*Cicer arietinum*); and *aalu arak* (*Solanum tuberosum*). Commonly consumed meat varieties included varieties like: pigeon, local name *perua* (*Columbia livia intermedia*); boar, local name *barha* (*Sus indicus*); mussel, local name *jhinuk* (*Margaritifera margaritifera*); snail, local name *ghongi* (*Pila globosa*); field rat, local name *moosa* (*Rattus argentiventer*); crab, local name *kenkda* (*Paratephusa spinigera*); and duck (*Anaspplatyrhyncha* spp.). Indigenous varieties of fruits included: *tarop* (scientific name not available); *tiril* (*Diospyros melanoxylon*); *dahu* (*Artocarpus lakoocha* Roxb.); *mahua* (*Madhuca latifolia*); and *layu* (scientific name not known). Indigenous varieties of vegetables included: *sem* (*Dolichos lablab*); *bada ghangra* (*Dolichos iat* Jang, Linn.); and *pindra* (scientific name not available). Commonly reported indigenous tubers included *kapu* (*Dioscorea bulbifera*). The majority of the HH reported that indigenous foods like GLV (65·6 %), vegetables (71·6 %), roots and tubers (66·9 %), fruits (73·2 %) and meat and meat products (67·1 %) were consumed by all family members irrespective of age and sex. Most of the HH also reported storing and preserving indigenous foods for consumption during lean periods and throughout the year. These included GLV like *aalu arak*, *but arak*, *gandhari*, *garundi arak*, *haisa arak*, *kaddu arak*, *kantha arak*, *matha arak*, *muli arak*, *pyaz arak*, mustard leaves, *sin arak* and *turi arak*; vegetables like brinjal and *sem*; fruits like mango and *ber*; and mushrooms. Methods of preservation included sun drying or boiling the vegetables followed by sun drying.

### Dietary intake pattern of adults in the households

Most of the HH interviewed in the study villages (90·0 %) were non-vegetarians. Most of the adults consumed two (51·0 %) or three (48·3 %) main meals daily. Consumption of in-between meals was reported by 68·2 %. The foods commonly consumed as in-between meals included both fresh and processed foods like puffed rice (87·4 %), rice flakes (60·2 %), fruits (75·7 %), biscuits (50·5), tea (44·7 %), *papad* (crisps made of lentil flour; 14·6 %) and others (14·6 %) like *dalmoth* (a savoury made of fried lentils), *maad bhaat* (rice soup), *pao roti* (bread) and *roti* (Indian bread). The majority of HH (99·3 %) consumed packaged iodized salt. Consumption of alcohol by any member of the family was reported by almost all HH (93·4 %).

Although a variety of indigenous foods were reportedly consumed, a detailed FFQ carried out during the main survey in the rainy season (June 2013) revealed poor consumption of these foods on a daily basis. The majority of HH (57·6 %) consumed rice as a staple more than twice daily. GLV were the most common group of food items besides rice in the habitual diet. Almost a quarter of the population consumed GLV once daily while the consumption of other vegetables was reported to be only once or twice weekly by a majority (37·7 %). Frequent and copious consumption of seasonal fruits from the trees within the villages was also reported. Almost a quarter of the community (23·2 %) reported the consumption of fruits more than twice daily during the month of the survey. Almost a third (33·8 %) of the community reported consuming flesh foods once or twice per week in the last month and only a quarter of the community reported monthly consumption of any kind of fish. About half of the community (46·3 %) did not consume any milk or milk products during the month of the survey while only a third (33·1 %) consumed pulses/legumes one or two times weekly. Oil consumption twice or more than twice daily was reported by the majority of HH (75·0 %), with most of them consuming mustard oil. Most HH reported sugar consumption (16·6 %) once or twice weekly. Consumption of many indigenous varieties of alcoholic drink was reported which included *taadi* (alcohol made from palm sap), *khajur taadi* (alcoholic drink made from dates), *mahua taadi* (alcohol made from *mahua*) and *handiya* (alcoholic drink made from rice). A quarter (27·8 %) of the HH reported consuming these alcoholic beverages once daily.

### Nutrient intakes of women of reproductive age

A two-day 24 h DR was conducted with one randomly selected woman per HH during the first survey in the rainy season (*n* 147). DR were also carried out in one-third of these women in winter (*n* 60) and summer (*n* 47) seasons. For the analysis of DR data, we could find the nutrient composition or carry out nutrient analysis for all foods except for three (one GLV, one unidentified food item, one fruit) that were reported in the DR of women. The food items for which we did not conduct nutrient analysis were reported in the DR of only two women each in winter and summer season. Table 2 provides a detailed description of the nutrient intakes of the study women in the three seasons. When the dietary intakes were compared with the Indian RDA for moderately active women, the adequacy of mean energy, protein, niacin and Zn intakes of the study population was more than 80 % in all seasons. However, the percentage adequacy for fat was below 60 %. For micronutrients, the percentage adequacy was below 60 % for Ca, folic acid, riboflavin and Zn among the study women (*n* 147) in the rainy season. Among the women who were followed up in winter (*n* 60), the percentage adequacy was below 60 % for Fe, Ca, folic acid, vitamin A, riboflavin and vitamin B_12_ while in summer (*n* 48) the percentage adequacy was below 60 % for vitamin A, vitamin C and vitamin B_12_. The NAR for Fe, Ca, folic acid, riboflavin and vitamin B_12_ was inadequate in more than 50 % of the study participants in all three seasons. The NAR for vitamin A and vitamin C was inadequate in more than half of the study population in both winter and summer seasons, while the NAR for thiamin was inadequate in 50 % of the study population in the winter season.

**Table 2:** Daily nutrient intakes of women of reproductive age (15–49 years) in the sampled households, Santhal tribal community of Jharkhand, India

		Nutrient intake, season 1 (rainy; *n* 147)				NAR, season 1 (*n* 147)						Nutrient intake, season 2 (winter; *n* 60)				NAR, season 2 (*n* 60)						Nutrient intake, season 3 (summer; *n* 48)				NAR, season 3 (*n* 48)					
						Inadequate		Fairly adequate		Adequate						Inadequate		Fairly adequate		Adequate						Inadequate		Fairly adequate		Adequate	
Nutrient	RDA^(^ ^31^ ^)^	Mean	sd	Median	% adequacy*	*n*	%	*n*	%	*n*	%	Mean	sd	Median	% adequacy*	*n*	%	*n*	%	*n*	%	Mean	sd	Median	% adequacy*	*n*	%	*n*	%	*n*	%
Energy (kJ)	9330†	8238	3255	7895	88·3	–		–		–		9393	3908	9401	100·7	–		–		–		10418	3912	10012	111·6	–		–		–	
Energy (kcal)	2230†	1969	778	1887	88·3	–		–		–		2245	934	2247	100·7	–		–		–		2490	935	2393	111·6	–		–		–	
Protein (g)	55·0	43·5	18·9	40·1	79·1	58	39·4	59	40·1	30	20·4	53·6	21·7	50·5	97·4	13	21·7	25	41·7	22	36·7	63·7	60·3	60·3	115·8	5	10·4	15	31·2	28	58·3
Fat (g)	At least 10 E%[Table-fn tab2fn1]	4·0	2·3	3·8	40·1							2·9	2·3	2·45	29·3							3·3	3·1	2·2	33·0						
Fe (mg)	21·0	13·9	18·9	10·1	66·2	**103**	**70**·**1**	30	20·4	14	9·5	10·1	6·2	7·9	48·1	**50**	**83**·**3**	6	10·0	4	6·7	13·7	12·7	10·5	65·2	**31**	**64**·**6**	11	22·9	6	12·5
Ca (mg)	600	325·3	578·1	212·0	54·2	**124**	**84**·**3**	9	6·1	14	9·5	273·3	410·6	177·5	45·5	**51**	**85**·**0**	6	10·0	3	5·0	379·1	481·9	201·0	63·2	**36**	**75**·**0**	5	10·4	7	14·6
Folic acid, total (μg)	200	85·8	65·9	59·8	42·9	**117**	**79**·**6**	16	10·9	14	9·5	78·9	42·8	72·5	39·4	**53**	**88**·**3**	7	11·7	0	0·0	111·1	94·7	82·0	129·5	**35**	**72**·**9**	8	16·7	5	10·4
Vitamin A (μg)	600	923·5	1364·8	428·0	153·9	59	40·2	12	8·1	76	51·7	94·7	204·7	4·0	15·8	**57**	**95**·**0**	0	0·0	3	5·0	54·8	138·8	0·0	9·1	**45**	**93**·**7**	2	4·2	1	2·1
Vitamin C (mg)	40	98·9	115·9	63·0	247·2	31	21·1	17	11·6	99	67·3	55·7	189·0	24·5	139·2	**31**	**51**·**7**	16	26·7	13	21·7	38·3	46·7	21·0	38·7	**28**	**58**·**3**	7	14·6	13	27·1
Thiamin (mg)	1·1	0·9	0·6	0·7	81·8	72	48·9	46	31·3	29	19·7	0·7	0·4	0·6	77·8	**42**	**70**·**0**	10	16·7	8	13·3	0·9	0·5	0·8	81·8	22	45·8	13	27·1	13	27·1
Riboflavin (mg)	1·3	0·6	0·3	0·6	47·7	**122**	**82**·**9**	18	12·2	7	4·8	0·5	0·2	0·4	38·5	**54**	**90**·**0**	5	8·3	1	1·7	0·5	0·2	0·5	38·5	**43**	**89**·**6**	4	8·3	1	2·1
Niacin (mg)	14	20·9	8·7	20·0	149·3	8	5·4	19	12·9	120	81·6	13·0	5·8	13·0	92·8	3	5·0	10	16·7	47	78·3	15·4	6·5	14·5	110·0	2	4·2	4	8·3	42	87·5
Zn (mg)	10	7·8	3·2	7·5	78·0	54	36·7	68	46·2	25	17·0	9·4	3·8	9·0	94·0	14	23·3	23	38·3	23	38·3	10·1	4·1	10·0	101·0	8	16·7	19	39·6	21	43·7
Vitamin B_12_ (µg)	1·0	0·18	0·14	0·0	18·0	**139**	**94**·**6**	1	0·7	7	4·8	0·1	0·3	0·0	10·0	**56**	**93**·**3**	1	1·7	3	5·0	0·02	0·1	0·0	2·0	**47**	**97**·**9**	0	0·0	1	2·1

NAR, nutrient adequacy ratio (**i**nadequate, <0·66 of the RDA; fairly adequate, 0·66 to <1·00 of the RDA; adequate, ≥1·00 of the RDA); E%, percentage of total energy. Bold font indicates that more than 50 % of the study population had inadequate intake of the specific nutrient.

* ‘% adequacy’ is the mean nutrient intake expressed as a percentage of the RDA, i.e. mean intake of nutrient/RDA of nutrient.

† RDA for moderately active women.

### Seasonal variation in median intakes of nutrients in women

In order to explore any seasonal variation in the nutrient intakes of the study population, thirty-seven women (a quarter of the study population) were followed in all three seasons for assessment of their dietary intake. Table 3 shows their median nutrient intakes in the three seasons.

**Table 3:** Seasonal variations in median daily nutrient intakes* of women of reproductive age (15–49 years) in the sampled households, Santhal tribal community of Jharkhand, India

	Season 1 (rainy)		Season 2 (winter)		Season 3 (summer)		
Nutrient	Median	IQR	Median	IQR	Median	IQR	*P* value
Energy (kJ)	8268	7050–9648	9401	7569–10125	9883	8146–13045	0·125
Energy (kcal)	1976	1685–2306	2247	1809–2420	2362	1947–3118	0·125
Protein (g)	43·7	35·2–53·8	51·1	43·4–64·8	60·8	47·9–77·4	0·002
Fat (g)	12·2	7·7–16·1	10·8	7·0–15·5	15·0	8·0–20·0	0·149
Ca (mg)	248·0	158·5–374	180·0	116·0–295·0	188·0	141·0–315·0	0·331
Fe (mg)	10·4	5·8–15·1	7·4	5·9–11·6	10·0	7·0–16·0	0·248
Zn (mg)	8·1	6·5–9·6	9·0	7·0–11·0	9·0	7·0–12·0	0·145
Vitamin C (mg) (*n* 36)	69·0	32·0–152·0	23·0	13·0–40·0	20·0	10·0–41·0	<0·001
Folate, total (μg)	69·3	42·3–153·2	72·0	41·0–85·0	82·0	58·0–135·0	0·028
Thiamin (mg)	0·8	0·5–1·3	0·6	0·5–0·8	0·8	0·5–1·2	0·004
Riboflavin (mg)	0·7	0·4–0·9	0·4	0·4–0·6	0·5	0·4–0·6	0·014
Niacin (mg)	12·7	11·0–16·4	13·0	11·0–15·0	14·0	12·0–18·0	0·060

IQR, interquartile range.

* Since most of the nutrient variables were not normally distributed, the median and IQR are presented. Test of comparisons used ANOVA on log-transformed variables. Comparisons of vitamin A and vitamin B_12_ intakes are not reported as many of the women had almost negligible intakes.

The intakes of protein (*P=*0·002), vitamin C (*P*<0·001), folic acid (*P*=0·028), thiamin (*P*=0·004) and riboflavin (*P*=0·014) were significantly different in the three seasons. The pairwise differences in nutrient intake between seasons are detailed in the online supplementary material, Table S1.

### Contribution of indigenous foods to nutrient intakes of women

In order to explore the contribution of indigenous foods to the nutrient intakes of the women surveyed, we compared the median nutrient intakes in women who had consumed any indigenous food in the past 2 d with those who had not. This comparison was done for the DR conducted during the rainy season only (*n* 147). Only 50 % of the study population consumed any kind of indigenous food during the recall period. The median intakes of commonly consumed indigenous foods under various food groups by those who reported consumption (*n* 74) are shown in Table 4. Although there were no differences in the median macronutrient intakes of women who consumed indigenous foods *v*. those who did not, the intakes of Ca (*P*=0·006) and Fe (*P*=0·010) were significantly lower in the group not consuming indigenous foods during the period of the DR (Table 5).

**Table 4:** Quantitative estimates of indigenous food intakes according to food group among those who reported consumption of indigenous foods in the past 2 d in the 24 h dietary recall (*n* 74); women of reproductive age (15–49 years), Santhal tribal community of Jharkhand, India

Food group	No. of women consuming the indigenous food group	Median intake of indigenous foods (g/d)	IQR (g/d)
Cereals	10	11·2	10·1–25·1
Pulses	31	17·8	11·0–22·5
GLV	29	33·5	15·9–55·0
Other vegetables	13	43·0	22·0–83·5
Fruits	15	35·7	10·0–67·5
Meat and meat products	1	50·0	50·0–50·0
Mushroom	2	11·7	8·3–15·0

IQR, interquartile range; GLV, green leafy vegetables.

**Table 5:** Comparisons of median daily nutrient intakes* between those who reported consumption of indigenous foods in the past 2 d in the 24 h dietary recall and those who did not; women of reproductive age (15–49 years), Santhal tribal community of Jharkhand, India

	Consuming indigenous foods (*n* 74)		Not consuming indigenous foods (*n* 73)		
Nutrient	Median	IQR	Median	IQR	*P* value
Energy (kJ)	7699	6238–9665	8268	6201–9485	0·980
Energy (kcal)	1840	1491–2310	1976	1482–2267	0·980
Protein (g)	42·1	31·7–51·3	38·9	31·1–52·8	0·753
Fat (g)	12·2	8·2–16·0	12·1	7·8–17·0	0·713
Ca (mg)	225·6	164·1–366·6	191·8	121·5–269·5	0·006
Fe (mg)	11·1	7·9–16·4	9·2	6·3–12·7	0·010
Zn (mg)	7·7	5·4–9·4	7·3	6·0–9·5	0·838
Vitamin C (mg)	67·6	33·6–125·1	58·2	29·5–119·6	0·299
Folate, total (μg)	53·2	34·0–103·8	73·8	40·2–121·5	0·065
Thiamin (mg)	0·7	0·6–1·1	0·7	0·5–0·9	0·318
Riboflavin (mg)	0·6	0·4–0·8	0·5	0·4–0·8	0·912
Niacin (mg)	20·3	15·8–24·9	19·8	16·0–24·6	0·639

IQR, interquartile range.

* Since most of the nutrient variables were not normally distributed, the median and IQR are presented. Test of comparisons used either the simple *t* test or the *t* test on log-transformed variables or Wilcoxon’s rank-sum test. Comparisons of vitamin A and vitamin B_12_ intake are not reported as many of the women had almost negligible intakes.

### Anthropometric assessment of women

Based on their weight and height, women (*n* 139) were classified under various categories of underweight using CED classification (Table 6). Almost half of the women (48·9 %) had various degrees of CED with about 14·7 % of the women falling in the category of CED III (BMI <16·0 kg/m^2^). This high level of CED among study women existed in spite of a mean energy adequacy of above 80 % in all three seasons. In order to assess any misreporting, we checked the data for any systematic bias towards over-reporting or under-reporting. This was done by applying Goldberg’s cut-off for energy intake to BMR for assessing any misreporting^(^ ^34^ ^)^. Based on the equation suggested, we calculated the PAL cut-offs at the upper and lower 95 % confidence limits and found the cut-offs to be 1·75 and 1·85, at a group PAL of 1·8 (moderately active women). The mean PAL for our study population was found to be 1·79, which was well within the acceptable limits. There was also no significant association (*P*=0·546) between indigenous food consumption and prevalence of underweight in women.

**Table 6:** BMI classification of the women of reproductive age (15–49 years), Santhal tribal community of Jharkhand, India

	Adult BMI classification							
	Underweight (BMI<18·5 kg/m^2^) (CED)		Increasing but acceptable risk* (BMI=18·5–23·0 kg/m^2^)		Increased risk* (BMI=23·0–27·5 kg/m^2^)		High risk*(BMI>27·5 kg/m^2^)	
	*n*	%	*n*	%	*n*	%	*n*	%
Women (*n* 139)	68	48·9	70	50·4	1	0·7	–	
	Various grades of CED classification							
	CED I		CED II		CED III			
	(BMI=17·0–18·4 kg/m^2^)		(BMI=16·0–16·9 kg/m^2^)		(BMI<16·0 kg/m^2^)			
	*n*	%	*n*	%	*n*	%		
Women (*n* 68)	44	64·7	14	20·6	10	14·7		

CED, chronic energy deficiency.

* The term ‘risk’ here denotes risk for overweight or obesity.

## Discussion

The Santhal community in the present study were small landholder farmers, where the majority of HH owned land for farming and for backyard gardening. The HH reported utilizing the produce from the agricultural field, *bari* and the land resources within and around the villages for household consumption. Other studies among Santhal tribes have also documented forest-dependent systems with the community utilizing products such as roots, tubers, firewood, etc. sometimes for subsistence and sometimes as supplements to their routine diet. Some of these forest products are also known to be particularly rich sources of energy and protein^(^ ^35^ ^)^. Indigenous foods such as some shellfish, mushrooms and plant seeds are consumed for their rich protein content. The regions inhabited by the Santhal community also have a number of genetically diverse rice varieties that are known to have better nutrient profile in terms of protein content, digestibility and micronutrient levels (e.g. niacin) as compared with routinely cultivated rice varieties^(^ ^36^ ^,^ ^37^ ^)^.

The high level of CED observed in the study community is of concern. Such levels of CED have also been reported in Santhal communities across different states of India^(^ ^17^ ^,^ ^38^ ^)^. Access to PDS as reflected by the possession of a ration card was low despite the high prevalence of CED and low food security in the community. Other studies have also documented a poor access to PDS among Santhals as well as socio-economic deprivation and nutritionally deficient diets in this community^(^ ^39^ ^–^ ^41^ ^)^.

Although many indigenous varieties of cereals, GLV, roots and tubers, other vegetables as well as fruits were reportedly consumed, the frequency of consumption was low. A typical day’s diet for a large majority consisted of rice and some kind of GLV that was procured from agricultural land, *bari* or forest. Poor consumption of protein-rich foods like pulses, milk and meat products was observed. Seasonal intake of fruits was observed in the survey that was conducted during the rainy season. Similar food intake patterns along with traditional food practices and use of a variety of edible weeds in this community have been reported earlier^(^ ^20^ ^,^ ^42^ ^)^.

The nutrient intake profile of the women showed poor intakes of various macro- and micronutrients. The mean intakes of some of the crucial nutrients like Fe, Ca and folic acid were inadequate in a large majority of the population during the three seasons. Poor protein intake has also been reported in the Santhal community in West Bengal^(^ ^38^ ^)^. Seasonal variations in intakes of many micronutrients were also observed. This could possibly be attributed to the availability of seasonal indigenous foods rich in these micronutrients at specific times of the year. Community-based programmes teaching women how to optimize the practices of preservation of foods for later consumption could be a suitable strategy in this scenario that may compensate for lower nutrient intakes during lean seasons.

Another significant study observation was that women consuming indigenous foods in the past 24 h had higher intakes of Fe and Ca than those who did not. This clearly indicates that indigenous foods might contribute to better intakes of crucial micronutrients like Fe and Ca provided they are routinely consumed. It has also been seen that regular consumption of indigenous varieties of fruits can potentially contribute to fulfilling dietary recommendations for vitamin A^(^ ^7^ ^,^ ^8^ ^)^. In addition, Ogle *et al*. found that the daily intake of some naturally occurring vegetables could fulfil up to 30 and 40 % of the recommended allowances for vitamin A and Ca, respectively^(^ ^9^ ^)^. Previous research and our findings both strongly support the premise that the indigenous foods identified in the present study can contribute to nutrition security and may address poor micronutrient intake or ‘hidden hunger’ in the Santhal community if optimally consumed and promoted for their nutritional benefits. Evidence from other research on indigenous food systems indicates that indigenous plants could facilitate improvement in health status of individuals in both food-secure and food-insecure HH. Individual case studies also document the success of traditional food systems in improving nutritional status in communities that could, in certain conditions, provide over half of daily dietary energy requirements^(^ ^43^ ^)^.

Thus our study indicates the potential of encouraging cultivation and/or regular consumption of nutrient-dense seasonally available indigenous foods to address dietary inadequacies in the study population. In this context, a model home garden plot has been developed for two districts of India, with different sets of cropping sequences that can provide vegetables for a family of four all year round^(^ ^44^ ^)^. Most of the existing national food security schemes focus on improving only macronutrient intakes (i.e. of energy, protein and fat), rather than a holistic approach to ensure adequate diversity of nutrients necessary for a healthy and productive life^(^ ^45^ ^)^. The present scenario demands a complementary strategy of availing schemes that promote food security along with optimizing the utilization of agricultural lands and *baris.* This could be done through efforts that increase the proportion of food procurement from the nearby environment, higher production of nutrient-dense indigenous foods and dissemination of knowledge on the nutritive value of these foods. This approach may prove to be a highly cost-effective strategy for increasing consumption and addressing undernutrition, micronutrient deficiency and food security in this community^(^ ^18^ ^,^ ^19^ ^)^.

### Limitations of the study

The 24 h DR were conducted for 2 d only in each season and estimating the percentage of inadequacy (<66 % of the RDA) using a 2 d DR may overestimate the true population prevalence of inadequacy.

Various foods for which we did not have nutritive values documented in the Indian food composition tables were stated to be consumed by women in 2 d of DR in three seasons. While efforts were made to carry out nutrient analysis of those foods in an NABL-certified laboratory using standard procedures and laboratory quality control and quality assurance practices as part of the present study, we could not get nutrient analysis done on three food items. These foods were consumed by two participants each in summer and winter season. Therefore, we expect some underestimation of nutrient intakes in those study participants.

The nutritive values of some of the food items used for dietary analysis were not published data and were analysed as part of the study. However, the findings on the nutritive values have been submitted as a manuscript to another journal and are currently under review.

The FFQ used in the study was developed and pre-tested as a part of the study. We did not use any validated Santhal food FFQ for the present study.

## Conclusion

The inadequate nutrient intakes observed in our study community exist amid an environment in which many nutrient-rich indigenous foods are available. The community has the traditional knowledge about the availability and access to these foods, but perhaps does not utilize these optimally because of lack of awareness regarding their nutritive value. Efforts in terms of effective nutrition education and behaviour change regarding the importance of these foods and promotion of the preferential cultivation of nutrient-dense plants can go a long way in promoting better micronutrient intakes in the Santhal community of Jharkhand.

## Supplementary material

For supplementary material accompanying this paper visit https://doi.org/10.1017/S1368980016000318. click here to view supplementary material

## References

[1] Anthropological Survey of India (2015) A note on the Series, People of India – Anthropological Survey of India. http://www.ansi.gov.in/people_india.htm (accessed April 2015).

[2] Misra S, Maikhuri RK, Kala CP et al. (2008) Wild leafy vegetables: a study of their subsistence dietetic support to the inhabitants of Nanda Devi Biosphere Reserve, India. J Ethnobiol Ethnomed 4, 15. 18510780 10.1186/1746-4269-4-15 PMC2430554

[3] Frison EA, Smith IF, Johns T et al. (2006) Agricultural biodiversity, nutrition, and health: making a difference to hunger and nutrition in the developing world. Food Nutr Bull 27, 167–179. 16786983 10.1177/156482650602700208

[4] Department of Social Development & Department of Agriculture, Forestry and Fisheries (2013) Annexure A. A National Policy on Food and Nutrition Security for the Republic of South Africa. http://www.daff.gov.za/docs/media/NATIONAL%20POLICYon%20food%20and%20nutrirition%20security.pdf (accessed July 2015).

[5] Bhattacharjee L, Saha SK & Nandi BK (2007) Food-Based Nutrition Strategies in Bangladesh: Experience of Integrated Horticulture and Nutrition Development. Bangkok: FAO Regional Office for Asia and the Pacific; available at http://www.fao.org/3/a-ag126e.pdf

[6] Roche ML, Creed-Kanashiroa HM, Tuestaa I et al. (2008) Traditional food diversity predicts dietary quality for the Awajún in the Peruvian Amazon. Public Health Nutr 11, 457–465. 17610756 10.1017/S1368980007000560

[7] Davey MW, Van den Bergh I, Markham R et al. (2009) Genetic variability in *Musa* fruit provitamin A carotenoids, lutein and mineral micronutrient contents. Food Chem 115, 806–813.

[8] Englberger L, Kuhnlein HV, Lorens A et al. (2010) Pohnpei, FSM case study in a global health project documents its local food resources and successfully promotes local food for health. Pac Health Dialog 16, 129–136. 20968245

[9] Ogle BM, Dao HT, Mulokozi G et al. (2001) Micronutrient composition and nutritional importance of gathered vegetables in Vietnam. Int J Food Sci Nutr 52, 485–499. 11570015 10.1080/713671806

[10] Singh V & Garg AN (2006) Availability of essential trace elements in Indian cereals, vegetables and spices using INAA and the contribution of spices to daily dietary intake. Food Chem 94, 81–89.

[11] Englberger L, Lyons G, Foley W et al. (2010) Carotenoid and riboflavin content of banana cultivars from Makira, Solomon Islands. J Food Compost Anal 23, 624–632.

[12] Bhattacharjee L, Kothari G, Priya V et al. (2009) The Bhil food system: links to food security, nutrition and health. In Indigenous Peoples’ Food Systems: The Many Dimensions of Culture, Diversity and Environment for Nutrition and Health, pp. 209–230 [HV Kuhnlein, B Erasmus and D Spigelski, editors]. Rome: FAO and Centre for Indigenous Peoples’ Nutrition and Environment; available at ftp://ftp.fao.org/docrep/fao/012/i0370e/i0370e.pdf

[13] Arnold F, Parasuraman S, Arokiasamy P et al. (2009) *Nutrition in India. National Family Health Survey (NFHS-3), India, 2005*–*2006* . Mumbai and Calverton, MD: International Institute for Population Sciences and ICF Macro; available at http://www.rchiips.org/nfhs/nutrition_report_for_website_18sep09.pdf

[14] Office of the Registrar General (2001) Jharkand Data Highlights: The Scheduled Tribes, Census of India 2001. India: Office of the Registrar General; available at http://censusindia.gov.in/Tables_Published/SCST/dh_st_jharkhand.pdf

[15] Dutta CS, Chakraboraty T & Ghosh T (2008) Prevalence of under nutrition in Santal children of Puriliya district West Bengal. Indian Pediatr 45, 43–46. 18250505

[16] Chakraborty U, Dutta CS, Dutta G et al. (2008) A comparative study of physical growth and nutritional status in Santal children of Ghatsila and Bolpur. Tribes Tribals 2, 79–86.

[17] Bose K, Chakraborty F, Mitra K et al. (2006) Nutritional status of adult Santal men in Keonjhar District, Orissa, India. Food Nutr Bull 27, 353–356. 17209478 10.1177/156482650602700410

[18] Chatterjee S, Dhar S, Sengupta B et al. (2011) Coexistence of haemoglobinopathies and iron deficiency in the development of anemias in the tribal population eastern India. Stud Tribes Tribals 9, 111–121.

[19] Rao T & Vijay T (2006) Malnutrition and anemia in tribal pediatric population of Purnia district (Bihar). Indian Pediatr 43, 181–182. 16528120

[20] Sinha R & Lakra V (2005) Wild tribal food plants of Orissa. Indian J Trad Knowl 4, 246–252.

[21] Longvah T & Deosthale YG (1998) Compositional and nutritional studies on edible wild mushroom from northeast India. Food Chem 63, 331–334.

[22] Longvah T & Deosthale YG (1998) Nutrient composition and food potential of *Parkia roxburghii*, a less known tree legume from northeast India. Food Chem 62, 477–481.

[23] Deosthale YG & Longvah T (1991) Chemical and nutritional studies on Hanshi (*Perilla frutescens*), a traditional oilseed from northeast India. J Oil Fat Ind 68, 781–784.

[24] Johns T & Eyzaguirrea PB (2006) Linking biodiversity, diet and health in policy and practice. Proc Nutr Soc 65, 182–189. 16672079 10.1079/pns2006494

[25] National Sample Survey Office, Ministry of Statistics & Programme Implementation, Government of India (2013) Key Indicators of Household Consumer Expenditure in India, NSS 68th Round, July 2011–June 2012. New Delhi: National Sample Survey Organisation; available at http://mospi.nic.in/Mospi_New/upload/KI-68th-HCE.pdf

[26] Grinnell RM & Unrau YA (2007) Social Work Research and Evaluation: Foundations of Evidence-Based Practice. Oxford: Oxford University Press.

[27] World Health Organization (2002) The World Health Survey. Sampling Guidelines for Participating Countries. http://www.micronutrient.org/nutritiontoolkit/ModuleFolders/5.Sampling/resources/WHO_sampling_guidelines.pdf (accessed June 2013).

[28] Blumberg SJ, Bialostosky K, Hamilton WL et al. (1999) The effectiveness of a short form of the Household Food Security Scale. Am J Public Health 89, 1231–1234. 10432912 10.2105/ajph.89.8.1231 PMC1508674

[29] Bickel G, Nord M, Price C et al. (2000) Guide to Measuring Household Food Security: Revised 2000. Alexandria, VA: US Department of Agriculture, Food and Nutrition Service; available at http://www.fns.usda.gov/sites/default/files/FSGuide.pdf

[30] Gopalan C, Sastri BV & Balasubramanian SC (2004) Nutritive Value of Indian Foods. Hyderabad: Indian Council of Medical Research. 14045465

[31] National Institute of Nutrition (2010) Nutrient Requirement and Recommended Dietary Allowances for Indians. A Report of the Expert Group of the Indian Council of Medical Research 2009. Hyderabad: National Institute of Nutrition; available at http://icmr.nic.in/final/RDA-2010.pdf

[32] Malhotra A & Passi SJ (2007) Diet quality and nutritional status of rural adolescent girl beneficiaries of ICDS in north India. Asia Pac J Clin Nutr 16, 8–16. 17392069

[33] WHO Expert Consultation (2004) Appropriate body-mass index for Asian populations and its implications for policy and intervention strategies. Lancet 363, 157–163. 14726171 10.1016/S0140-6736(03)15268-3

[34] Black AE (2000) Critical evaluation of energy intake using the Goldberg cut-off for energy intake:basal metabolic rate. A practical guide to its calculation, use and limitations. Int J Obes Relat Metab Disord 24, 1119–1130. 11033980 10.1038/sj.ijo.0801376

[35] Food and Agriculture Organization of the United Nations (2014) *Indigenous Knowledge and Biodiversity*. *FAO Corporate Document Repository* . Rome: FAO; available at http://www.fao.org/docrep/004/v1430e/V1430E03.htm

[36] Rai R & Nath V (n.d.) The role of ethnic and indigenous people of India and their culture in the conservation of biodiversity. http://www.fao.org/docrep/article/wfc/xii/0186-a1.htm (accessed October 2015).

[37] Indo-Global Social Service Society (n.d.) Mushroom Cultivation – Ensuring Food Security for Tribal Families. http://igsss.org/newsevents/mushroom-cultivation-ensuring-food-security-for-tribal-families (accessed October 2015).

[38] Ghosh S (2014) Deficiency and sources of nutrition among an Indian tribal population. Coll Antropol 38, 847–853. 25420365

[39] Gupta S (2013) Poor and state entitlements: a case study of selected tribes of Jharkand. Jharkhand J Soc Dev 5, 1–7; available at http://www.iesd.org.in/jjsd/Journal%20pdf/2013-V-1&2%20Smita%20Gupta.pdf

[40] Saha KB & Saha U (1998) An overview of the socio-economic and demographic transition among the Santal: a census analysis. Man India 78, 87–101. 12294007

[41] Moitra A & Choudhary RP (1991) Food habits and anthropometry of two tribes of Rajmahal hills, Bihar. *Indian J Med* Res 94, 64–70. 2071187

[42] Ghosh S & Malik S (2008) Assessment and administration of health in a tribal community of India. Internet J Biol Anthropol 3, issue 2; available at http://ispub.com/IJBA/3/2/7040

[43] Durst P & Bayasgalanbat N (editors) (2014) *Promotion of Underutilized Indigenous Food Resources for Food Security and Nutrition in Asia and the Pacific* . Bangkok: FAO Regional Office for Asia and the Pacific.

[44] Keatinge JD, Chadha ML, Hughes J et al. (2012) Vegetable gardens and their impact on the attainment of the millennium development goals. Biol Agric Hortic 28, 1–15.

[45] Graham RD, Welch RM, Saunders DA et al. (2007) Nutritious subsistence food systems. Adv Agron 92, 1–74.

