# Supplementary material for: Contribution of indigenous foods towards nutrient intakes and nutritional status of women in the Santhal tribal community of Jharkhand, India
Source: Public Health Nutr. 2016 Mar 16;19(12):2256–67. doi: 10.1017/S1368980016000318 (PMC4988270; doi:10.1017/S1368980016000318)
Supplement: Supplementary file 1 [file S1368980016000318sup001.docx]

Table S1. Seasonal variations in nutrient intake: Multiple comparison P-values after Bonferroni correction (corrected P-value of 0.02)

| Nutrient | Season 1 vs. Season 2 | Season 2 vs. Season 3 | Season 3 vs. Season 1 |
| --- | --- | --- | --- |
| Protein | 0.041 | 0.003 | 0.001 |
| Vitamin C | <0.001 | 0.969 | <0.001 |
| Folate | 0.039 | 0.009 | 0.531 |
| Thiamine | 0.006 | 0.011 | 0.798 |
| Riboflavin | 0.017 | 0.453 | 0.025 |
